# Adiposity Rebound or Fat-Free Mass Anabolism in Children—Challenging a 42-Year-Old BMI Puzzle with Waist-to-Height Ratio: The ASNF-NNF 2025 Inaugural Flemming Quaade Award for Innovation in Childhood Obesity Lecture

**DOI:** 10.1016/j.tjnut.2026.101437

**Published:** 2026-03-10

**Authors:** Andrew O Agbaje

**Affiliations:** 1 Institute of Public Health and Clinical Nutrition, School of Medicine, Faculty of Health Sciences, University of Eastern Finland, Kuopio, Finland; 2 Children’s Health and Exercise Research Centre, Department of Public Health and Sports Sciences, Faculty of Health and Life Sciences, University of Exeter, Exeter, United Kingdom

**Keywords:** childhood obesity, body composition, metabolic health, muscle mass, prevention

## Abstract

**Background:**

Rolland-Cachera et al. introduced the concept of “adiposity rebound” in a paper published in the *American Journal of Clinical Nutrition* in 1984. They observed that body mass index (BMI) increased during the first year of life and then decreased with a renewed rise at about age 6 y, which they termed “adiposity rebound,” concluding that early rebound increased the risk of excess adiposity in later years. Although this concept has been vigorously criticized, an alternative explanation for this phenomenon has been lacking for 42 y. Moreover, BMI does not distinguish between fat mass and muscle mass.

**Objectives:**

To examine whether a more accurate surrogate measure of adiposity, waist circumference to height ratio (WHtR), confirms or refutes BMI-based adiposity rebound.

**Methods:**

In this study, 2410 children and adolescents’ data aged 2–19 y from the US National Health and Nutrition Examination Survey 2021–2023 cycle were analyzed using a more accurate WHtR. Both raw values of BMI and WHtR and their z-scores were plotted to ascertain the trajectory of adiposity with increasing age.

**Results:**

The mean value of BMI at age 2 y (17.1 kg/m_2_) was regained by age 6 y (mean BMI 17.0 kg/m_2_), while the mean BMI at age 7 y was 17.3 kg/m_2_ after a significant decrease (adiposity rebound). The WHtR mean value at age 2 y (0.54) was never regained throughout childhood and adolescence (0.51). Although BMI-adiposity rebound seems to be completed by age 6 y, WHtR, which specifically assesses fat mass, continued decreasing. A body composition reset (BCR) at the intersection of BMI and WHtR trajectories at age 4 y until WHtR nadir at age 7 y was observed. The BCR is a post-infancy BMI increase after an initial decline that simultaneously corresponds to a continued WHtR-adiposity physiologic decrease, culminating at the lowest WHtR trajectory before a subsequent WHtR increase.

**Conclusions:**

These novel findings establish that BMI-adiposity rebound is not physiologic but an epiphenomenon. I posit that “adiposity rebound” is a BMI-induced false discovery similar to the “obesity paradox” in adults. Therefore, fat-free mass or skeletal muscle mass anabolism is likely the accurate physiologic explanation for the BCR effect that occurs in early childhood.

## Introduction

“*Now our knowledge is partial and incomplete, and even the gift of prophecy (science) reveals only part of the whole picture!” 1 Corinthians 13:9 NLT*.

Forty-two years ago, French scientists Rolland-Cachera et al. [1] introduced the concept of “adiposity rebound” in a paper published in the *American Journal of Clinical Nutrition* in 1984. This seminal work has been cited >1300 times. The authors predicted the evolution of adiposity during growth, where individual adiposity curves, assessed by the weight/height^2^ (BMI), were plotted among 151 children aged 1 mo to 16 y [1]. They found that BMI-assessed adiposity increased during the first year of life and then decreased. The authors observed a renewed rise at about age 6 y, which they termed “adiposity rebound” [1]. They concluded that there was a relationship between the age at adiposity rebound and final adiposity such that an early rebound (before 5.5 y) was followed by a significantly higher adiposity level than a later rebound (after 7 y), with a supporting explanation of the cellularity of adipose tissue [1]. The flaws with BMI assessment of adiposity have been universally established; it is therefore important to reevaluate the “adiposity rebound” phenomenon in the light of current evidence [2]. This discussion was part of the American Society for Nutrition Foundation (ASNF) - Novo Nordisk Foundation (NNF)’s inaugural Flemming Quaade Award Lecture for Innovation in Childhood Obesity, presented by the author at NUTRITION 2025, in Orlando, Florida, United States.

### Challenges with BMI assessment of adiposity

Although BMI continues to be employed universally in clinical guidelines, screening, and management of pediatric obesity, there are significant limitations that remain unresolved for decades [3]. These limitations include sex, age, and ethnic or racial variations, inability to distinguish fat mass from lean mass, and overestimation or underestimation of overweight and obesity prevalence [[2], [3], [4]]. With the emergence of large-scale epidemiologic studies that quantified adiposity with dual-energy X-ray absorptiometry (DXA), previous associations of higher BMI with higher vascular stiffness and carotid wall thickness, explained as pathologic obesity-induced vasculopathies in a generally living pediatric population, are being reinterpreted as physiologic vascular remodeling largely induced by increased lean mass but not fat mass [[5], [6], [7], [8]]. Moreover, the DXA-direct measure of body composition identified increased lean mass metabolic health benefits in decreasing the risk of excess adiposity, insulin resistance, and type 2 diabetes in both young and adult populations, previously obscured by BMI assessment [9,10]. Evidence from the United Kingdom suggests that children have 4-fold more lean mass than fat mass, which decreases to 3-fold by mid-twenties, making adiposity estimates using BMI spurious in relation to obesity diagnosis [4,8,11]. It is therefore important to reflect on what aspect of a child’s body composition grows rapidly within the first 7 y of life: muscle or fat? Can BMI identify this specifically? Is it related to fat mass? The answer to these questions helps clarify what several colleagues have postulated as “adiposity rebound” for 4 decades.

### Controversies regarding the BMI-adiposity rebound critical period in children

Twenty-five years ago, William Dietz [12], in his commentary published in the *Lancet,* titled “*adiposity rebound: reality or epiphenomenon?”* highlighted a few concerns, viz, “the lack of evidence both that the rebound in BMI is attributable to body fat and that the increased BMI observed in adults who have had early adiposity rebound is associated with an increase in body fat.” Dietz [12] argued that since the children with higher BMIs had an earlier rebound, this could represent an epiphenomenon, since it was unknown whether age at rebound or the BMI at rebound had a greater influence on adult BMI, independent of the time of rebound. Dietz [12] noted that the correlations between BMI at age 7 and adult BMI and between age at rebound and adult BMI were similar. It was unclear at the time whether increased BMI represented children who were heavier or had a reduced height velocity compared with children with a later increase in BMI. Dietz [12] concluded that measurement error in assessing BMI could confound early BMI rebound in children, such that a 2 cm error in height could introduce a BMI error of 0.5 kg/m^2^ if the child weighs 20 kg, and a retrospective identification of early adiposity rebound makes it difficult to prevent.

**FIGURE 1:**
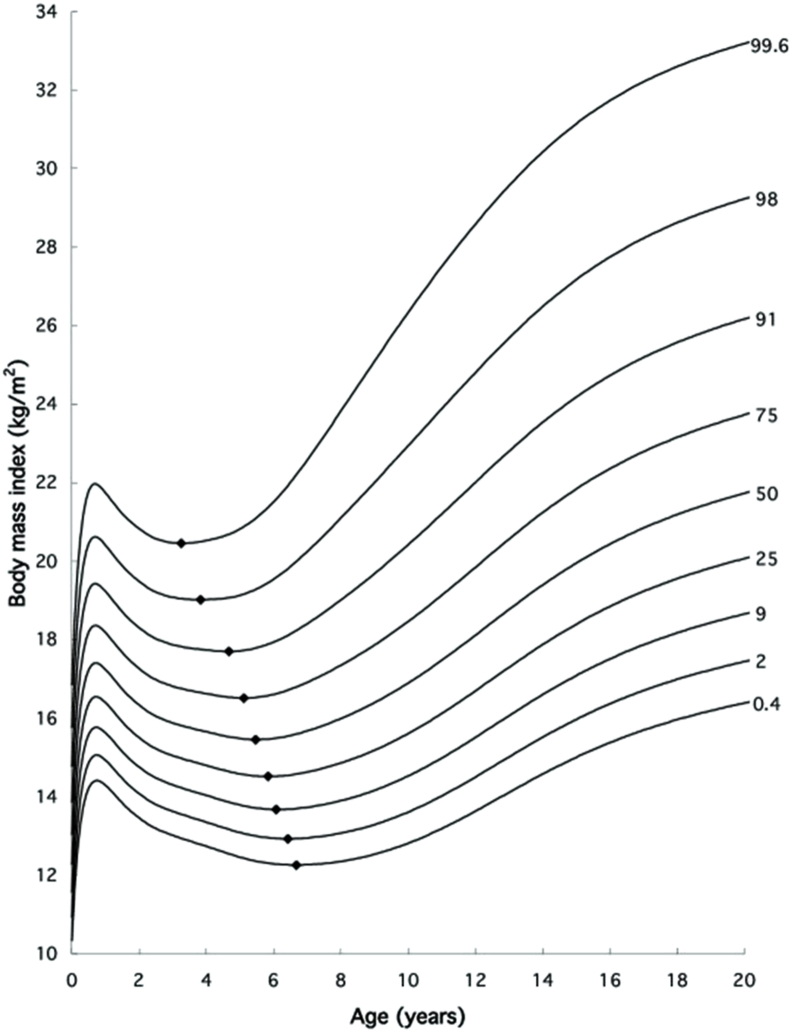
British 1990 girls BMI chart from Cole [13]. The age at adiposity rebound is inversely related to the BMI centile. Reproduced with permission from reference [13].

About 4 y later, Tim Cole [13] provided additional evidence (Figure 1) concluding “that the connection between early rebound and later fatness is not physiological but statistical.” Cole [13] noted that an early rebound is simply an upward centile crossing “horse racing effect.” This upward centile crossing predicts later obesity at whatever age it occurs, not just at rebound, i.e, the period is not bounded. Thus, the age at adiposity rebound fails to qualify as a critical period. A critical period ought to be “a developmental stage in which physiologic alterations increase the later prevalence of obesity. It needs *1)* a physiological alteration, and *2)* a bounded period with a distinct beginning and end” [12,13]. Nonetheless, the mechanism that drives an upward BMI centile crossing remains unknown [13]. A study reported that high protein intake was not associated with early *adiposity rebound,* but parental obesity increased the likelihood [14]. In another study, birth weight was similar in children classified as having very early, early, or later *adiposity rebound* [15]. A study identified higher genetic susceptibility to obesity score, small for gestational age and parental education as risk factors for early *adiposity rebound* [16]. On the contrary, evidence suggests that preterm infants have low levels of body fat at birth, whereas BMI is of limited use as a measure of body fatness in infancy and childhood [17,18].

### Critical surrogate and universal measure of adiposity—waist circumference-to-height ratio

Waist circumference-to-height ratio (WHtR) was first described by Ashwell in 1996 as a superior measure for assessing visceral and central adiposity compared with waist circumference, BMI and waist-to-hip ratio due to strong correlations with intra-abdominal fat measured with a computed tomography scan [[19], [20], [21], [22]]. Since then, WHtR has been related to several health outcomes, including mortality [2,[23], [24], [25]]. The United Kingdom is the first and only country to date to publish an obesity clinical guideline, the National Institute for Health and Care Excellence (NICE) guideline, which recommended the diagnosis of obesity by assessing WHtR in both adults and children [26]. Several WHtR cutpoints have been proposed for predicting increased cardiometabolic risks. However, these WHtR cutpoints were largely based on ease of memorization rather than rigorous statistical methods [[19], [20], [21],^26^]. For example, the United Kingdom NICE guideline recommends central adiposity WHtR classification in adults and children and youth as follows: “healthy central adiposity: WHtR 0.40 to 0.49, indicating no increased health risk; increased central adiposity: WHtR 0.50 to 0.59, indicating increased health risk, and high central adiposity: WHtR ≥0.60, indicating further increased health risk.” Dr Ashwell [27] who served on the NICE clinical guideline writing committee on overweight and obesity management and significantly influenced the adoption of WHtR stated in a published article regarding the WHtR cutpoint beyond 0.50 that “the other boundary values were determined on the basis of common sense.” Adopting such subjective bias into the NICE clinical guideline weakens its internal and external validity, making it unscientific and grossly misleading. A rigorous methodological study in adults conducted in 2013 concluded that the best predictor of both fat mass percentage and visceral adipose tissue fat mass in males and females was WHtR with derived cut points of 0.53 in males and 0.54 in females [22].

Pediatric research usually lags behind adult evidence and suffers from the superimposition of adult discoveries on the young population without acknowledging the significant differences in the body composition of growing children and adolescents. The United Kingdom’s WHtR value of >0.6 for detecting excess health risks is rare in the general pediatric population; moreover, the range of 0.5–0.6 is excessively wide, such that it accommodates both slightly increased fat and excessively high fat mass in a pediatric population [4,11]. In 2024, a large-scale longitudinal study of >7000 children from the Avon Longitudinal Cohort of Parents and Children, in England, United Kingdom, followed up for 15 y, identified that WHtR is the best predictor of DXA-measured total body fat mass and trunk fat mass (∼90% absolute agreement) [11]. The study also reported that WHtR does not vary with age and distinguishes fat mass from lean mass since WHtR had very low absolute agreement with lean mass (∼20%) [11]. These WHtR characteristics overcame several limitations of BMI. The following WHtR cutpoints were derived 0.40 to <0.50 in males, 0.4 to <0.51 in females as estimated normal fat mass; 0.50 to <0.53 in males, 0.51 to <0.54 in females, as high fat mass adiposity (visceral fat and total fat), which potentially corresponds to BMI overweight; and ≥0.53 (males)/≥0.54 (females) as excess fat mass adiposity (visceral fat and total fat) that potentially corresponds to BMI obesity [11]. Physiologically, females have more fat mass than males; hence, a single value WHtR cutpoint >0.5 for both sexes is inaccurate and could lead to misdiagnosis, mislabeling and mental health adversities. The new pediatric WHtR cutpoints have been validated as predictors of type 2 diabetes, liver steatosis and fibrosis, and bone fracture in multiracial adult populations, performing significantly better than BMI [4,11,28,29]. Recent randomized clinical trials among adults have confirmed the near-universal applicability of WHtR as a surrogate measure of adiposity across major racial groups in the world [24,30,31]. A recent large-scale cross-cohort study of Finnish adults (*n* = 4419) and United Kingdom adults (*n* = 450,896) concluded that “Neither BMI nor waist-to-hip ratio on its own exceeded the accuracy of WHtR, while WHtR was a better predictor of type 2 diabetes and heart disease than either of the 2 alone” [32]. Taken together, WHtR could be the ideal inexpensive and universal replacement for BMI in accurately diagnosing obesity globally, thereby addressing a knowledge gap highlighted for future research in the 2023 American Academy of Pediatrics obesity clinical practice guideline [3].

### Continued complexities with the BMI-obesity paradox in adults’ cardiovascular diseases

Another BMI-induced misleading terminology is the *obesity paradox*. This was first proposed in 2002 by Gruberg et al. [33] who described an unexpected finding that patients with overweight and obesity undergoing percutaneous coronary intervention had lower mortality rates than their normal-weight counterparts. Four years later, the “obesity paradox” appeared to be confirmed in a large meta-analysis involving 250,000 patients with coronary artery disease, where BMI >35 kg/m^2^ was not associated with an increase in total mortality [34]. The seemingly cardioprotective effect of high BMI, its biological hypotheses and mechanisms, and the limitations of the studies that identified the U-shaped BMI relationship with cardiovascular diseases (CVDs) were well summarized by Donini et al. [35]. In a 2013 debate on the nonexistence of the “obesity paradox,” Standi et al. [36] argued that selection bias, treatment bias, distinct phenotypes of patients with CVD, differences in prognosis, presence of comorbidities and confounders, anabolic deficiency or the malnutrition-inflammation syndrome could explain the reverse epidemiology, highlighting the need for randomized controlled trials.

Since 2023, several randomized controlled trials have compared BMI to WHtR in predicting heart failure [25,31]. These trials also identified the U-shaped BMI associations with CVD risks; however, WHtR, a better surrogate measure of excess adiposity, had a linear relationship with CVD risks [25,31]. For example, the Prospective Comparison of ARNI [angiotensin receptor-neprilysin inhibitor] with ARB [angiotensin-receptor blockers] Global Outcomes in Heart Failure with Preserved Ejection Fraction (PARAGON-HF) trial randomly assigned 4796 patients with heart failure, and post hoc analysis evaluated how BMI or WHtR predicted heart failure [25]. In contrast to BMI, U-shaped associations, WHtR was linearly associated with heart failure outcomes, and identified a higher proportion of patients who had a particularly elevated risk (i.e., ≥30%), and was not accompanied by evidence of an “obesity paradox.” The authors concluded that future trials of weight loss interventions in heart failure with preserved ejection fraction should identify eligible patients by WHtR, and not BMI and that “obesity paradox” does not exist [25].

A recent participant-level pooled analysis of 5 international randomized controlled trials that enrolled 21,479 adults concluded that although BMI exhibited complex J- and U-shaped associations with heart failure outcomes, higher WHtR was linearly associated with increased risk of heart failure and mortality events [24]. The study also noted that WHtR identified a substantial number of individuals with visceral obesity despite having BMI of <30 kg/m^2^, which may enhance risk stratification beyond BMI alone in heart failure diagnosis and management [24]. These discoveries about WHtR estimated fat mass-driven heart failure pathologies from randomized controlled trials have led to the novel adipokine hypothesis of heart failure with a preserved ejection fraction published by the *American College of Cardiology* in 2025 [24,25,30]. The novel theory emphasizes that heart failure with a preserved ejection fraction evolves as an adipose-driven derangement that is disseminated through endocrine-paracrine signaling to the heart [30].

### Consensus statements and frameworks on redefining obesity in adults with WHtR

In 2024, the *European Association for the Study of Obesity* (EASO) published a framework which acknowledged that the diagnosis of obesity was still based solely on BMI cutoff values, which does not reflect the role of adipose tissue distribution and function in the severity of the disease [23]. EASO explicitly stated that abdominal (visceral) fat accumulation is an important risk factor for health deterioration, even in the presence of low BMI and asymptomatic clinical manifestations [23]. EASO introduced WHtR, instead of waist circumference, in the obesity diagnostic process due to its superiority as a cardiometabolic disease risk marker [23]. The EASO recommended that excessive fat accumulation, which may confer an increased health risk in European adults, should be diagnosed with BMI ≥25 kg/m^2^ and a WHtR >0.5, representing a paradigm shift in obesity staging and diagnosis [23].

In 2025, the *Lancet Commission* issued a consensus statement on obesity definition and diagnosis, agreed on by 58 multinational and multispeciality experts [2]. The *Lancet Commission* recommended that “BMI should be used only as a surrogate measure of health risk at a population level, for epidemiological studies, or for screening purposes, rather than as an individual measure of health. Excess adiposity should be confirmed by either direct measurement of body fat such as DXA, where available, or by at least one anthropometric criterion (e.g., waist circumference, waist-to-hip ratio, or WHtR) in addition to BMI, using validated methods and cutoff points appropriate to age, gender, and ethnicity.” [2]. However, BMI of >40 kg/m^2^ does not require further excess adiposity confirmation as it can be safely assumed to be deleterious [2].

### Compelling evidence to refute “adiposity rebound” with “fat-free mass anabolism”

The age at *adiposity rebound* is defined as the age, beyond infancy, when an individual’s BMI is at a minimum [1]. First coined by Rolland-Cachera et al. [1] in 1984, while predicting the evolution of adiposity during growth in 151 children recruited from the age of 1 mo to 16 y. BMI-assessed adiposity increased during the first year of life and then decreased with a renewed rise at about age 6 y, which Rolland-Cachera et al. [1] described as a rebound of adiposity. Having detailed the obesity experts’ criticism of this epiphenomenon above, I now present a novel hypothesis that could better inform us of this unresolved puzzle. We now know that “obesity paradox,” as a cardioprotective effect of high BMI in adults does not exist because it is a misdiagnosis caused by BMI’s concurrent measurement of both fat mass and muscle mass [2,24,25,30]. Because WHtR assesses only fat mass but not muscle mass, I hypothesize that *adiposity rebound* in the pediatric population will cease to exist if WHtR is employed as a surrogate measure of adiposity rather than BMI [11]. To prove the hypothesis, I analyzed data from the multiracial United States NHANES 2021–2023 cycle data.

## Methods

This study included participants from the NHANES, which is a United States nationally representative study conducted by the National Center for Health Statistics of the Centers for Disease Control and Prevention to assess the health and nutritional status of adults and children in the United States. The study design and methods have been described in detail previously (https://www.cdc.gov/nchs/nhanes/about/index.html). The survey protocol was approved by the Research Ethics Review Board of the National Center for Health Statistics. Written informed consent from all participants was obtained by NHANES. This cohort study included 2492 children and adolescents from age 2 to 19 y who had complete BMI assessments during the most recent NHANES cycle conducted during the COVID-19 pandemic from August 2021 to August 2023. However, only 2410 children had both WHtR and BMI complete data. The original data generated and analyzed during this study are included in the Center for Diseases Control and Prevention data repository [37].

### Anthropometry

NHANES collected height, weight, and waist circumference via the Mobile Examination Center by trained health technicians. The participant’s age at the time of the screening interview determined the body measures examination protocol. BMI was calculated as weight in kilograms divided by height in meters squared. For participants aged <19 y, the Centers for Disease Control growth chart of >85th to <95th percentile was categorized as overweight and ≥95th percentile as obesity. WHtR was calculated as waist circumference in centimeters divided by height in centimeters. WHtR cut points are <0.40 (low fat mass), 0.40 to <0.50 (normal fat mass), 0.50 to <0.53 (high fat mass), and ≥0.53 (excess fat mass) in males. WHtR cut points for females are <0.40 (low fat mass), 0.40 to <0.51 (normal fat mass), 0.51 to <0.54 (high fat mass), and ≥0.54 (excess fat mass) [4,11,28]. An online tool for calculating WHtR specific to age and sex in the pediatric population is now available for clinical, public health, and research use at https://urfit-child.com/waist-height-calculator/.

### Statistical analysis

Participants’ descriptive characteristics were summarized as means and SD or frequencies and percentages. The differences in BMI and WHtR categories were estimated by the Pearson χ^2^ test of independence, and differences with *P* values <0.05 were considered statistically significant. The assumptions for the χ^2^ analyses include presenting data in frequencies, or counts, ensuring the categories are mutually exclusive, and each participant contributes data to only 1 cell, the study group being independent, and the value of the cell expected is ≥5 in approximately three-quarters of the cells [38]. Lastly, the mean values of BMI and WHtR trajectories were plotted at each age to either confirm or refute “adiposity rebound” theory. Z-score trajectories were also presented to allow adjustment for population regression to the mean, which could help distinguish a statistical phenomenon from a biological phenomenon (catch-up growth) [18].

## Results and Discussions

Altogether, 2492 children aged 2–19 y with complete BMI variables and 2410 with complete WHtR variables were included. The study population mean (SD) for BMI is 21.1 kg/m^2^, and WHtR is 0.497 (0.08). The frequency and prevalence of BMI classified underweight are 92 (3.7%), normal weight is 1469 (58.9%), overweight is 372 (14.9%), and obesity is 559 (22.4%). The frequency and prevalence of WHtR estimated fat mass categories are low fat mass 136 (5.6%), normal fat mass 1295 (53.7%), high fat mass 308 (12.8%), and excess fat mass 671 (27.8%). Altogether 50.6% of children and adolescents classified as BMI underweight had normal fat mass estimated with WHtR (Table 1). Also, 10% and 3.5% of children and adolescents categorized as BMI normal weight had high fat and excess fat mass estimated with WHtR, respectively (Table 1). A significant one-third of children and adolescents classified as BMI overweight have normal fat mass estimated with WHtR, whereas another one-third have excess fat mass (Table 1). Altogether, 90% of children and adolescents classified as having BMI obesity have excess fat mass estimated with WHtR.

**TABLE 1:** Cross-tabulations of BMI and WHtR categories in 2410 participants aged 2–19 y from the United States NHANES cohort

*n* = 2410	WHtR Low fat	Normal fat	High fat	Excess fat	Total
BMI underweight	42	44	1	0	87
Normal weight	94	1137	143	50	1424
Overweight	0	103	122	134	359
Obesity	0	11	42	487	540

The differences in categories were statistically significant *P* < 0.001.

Abbreviation: WHtR, waist circumference-to-height ratio.

**FIGURE 2:**
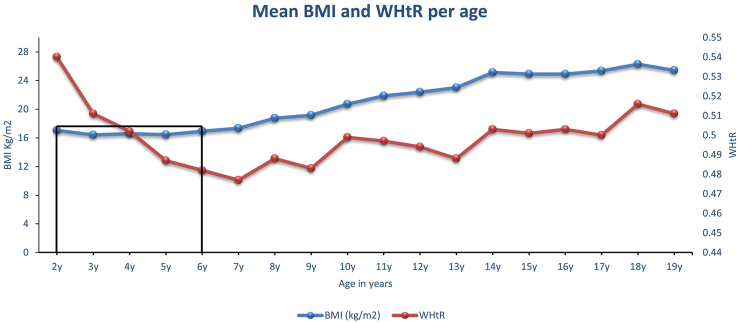
The NHANES 2021–2023 cycle, including 2–19 y olds (*n* = 2410). Supposed BMI-adiposity rebound in black was observed at age 2 y when mean BMI was 17.1 kg/m^2^ and at age 6 y when mean BMI was 17.0 kg/m^2^. WHtR, waist circumference-to-height ratio.

Next, the mean values of BMI and WHtR were plotted per age (Figure 2 and Table 2). BMI-based *adiposity rebound* appears to be confirmed since BMI at age 2 y was 17.1 and 17.0 kg/m^2^ at age 6 y, with a significant reduction of BMI between ages 3 and 5 y.

**TABLE 2:** Mean values of WHtR and BMI based on age in the United States NHANES 2021–2023 cohort *(n* = 2410*)*

Age (y)	2	3	4	5	6	7	8	9	10	11	12	13	14	15	16	17	18	19
BMI (kg/m^2^)	17.05	16.42	16.59	16.43	16.93	17.34	18.76	19.17	20.73	21.88	22.37	23.01	25.12	24.89	24.90	25.36	26.29	25.42
WHtR	0.540	0.511	0.502	0.487	0.482	0.477	0.488	0.483	0.499	0.497	0.494	0.488	0.503	0.501	0.503	0.500	0.516	0.511

The differences in categories were statistically significant *P* < 0.001.

Abbreviation: WHtR, waist circumference-to-height ratio.

**FIGURE 3:**
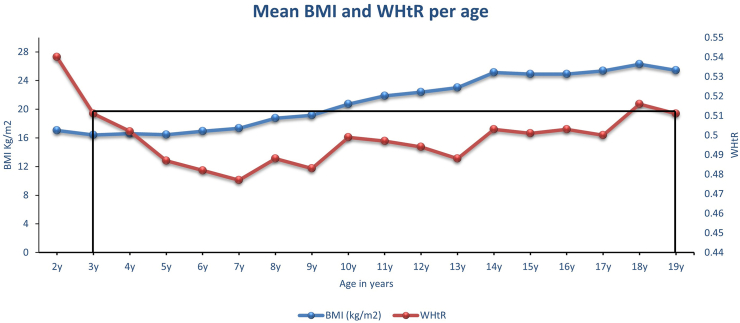
The NHANES 2021–2023 cycle, including 2–19 y olds (*n* = 2410). WHtR assesses both total and trunk fat (adiposity) but not muscle mass. WHtR at age 2 y was 0.54, and WHtR at age 18 y was 0.52. WHtR-adiposity rebound in black does not exist until after at least a 16-y period. WHtR at age 3 y was 0.51 and WHtR at age 19 y was 0.51. WHtR, waist circumference-to-height ratio.

**FIGURE 4:**
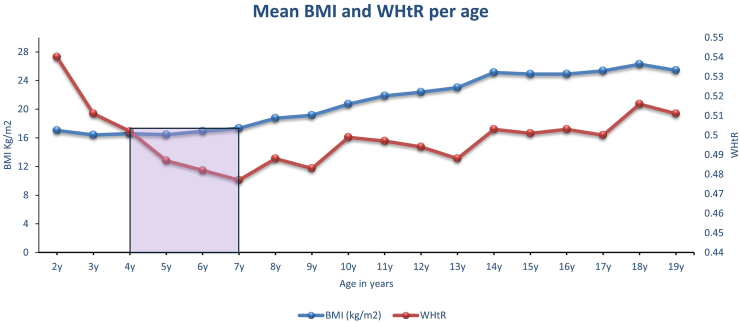
Mismatch between BMI-adiposity rebound and WHtR-adiposity normal physiologic trajectory is suggestive of a fat-free mass anabolism. Evidence from NHANES 2021–2023 survey cycle, including 2–19 y olds (*n* = 2410). WHtR, waist circumference-to-height ratio.

It is important to note that although BMI-*adiposity rebound* has occurred by age 6 y and thereafter increased linearly until late adolescence, WHtR estimated fat mass continued to decrease from ages 2 through age 7 y (Figures 2 and 3 and Table 2). This raises the question about what body composition indices underwent a rebound—fat mass or muscle mass? BMI assesses both muscle mass and fat mass, whereas WHtR specifically assesses fat mass [11]. Figure 4 shows that the mean WHtR at age 2 y was 0.54 and WHtR at age 18 y was 0.52. This suggests that if WHtR-adiposity rebound exists, it will take ∼16 y since the mean WHtR at age 3 y was 0.51 and the mean WHtR at age 19 y was 0.51 in the NHANES 2021–2023 cycle cohort.

The mismatch between BMI-adiposity rebound and WHtR-adiposity dynamic physiologic trajectory might suggest a “Body Composition Reset (BCR) effect,” which is a consequence of the metabolic changes during transition from intrauterine life and early extrauterine life (FIGURE 2, FIGURE 3). This BCR effect appears to set the stage for the next growth trajectory, which I will describe as “Fat-Free Mass Anabolism or Skeletal Muscle Mass/Lean Mass Anabolism.” Figure 4 shows that BCR effects occur around an mean age of 4 y, at the intersection of WHtR and BMI trajectories obtained from raw mean values. The BCR could be defined as the postinfancy BMI increase that corresponds to a WHtR-adiposity physiologic decrease, which culminates at the lowest WHtR mean value before a subsequent WHtR increase. The BCR transition period around ages 4–7 y might be a critical physiologic window for healthy muscle growth and future pubertal development. This critical period (BCR) might be the process of selecting an optimal body composition representation available from among the several competing factors that continually bombard the maturing musculoskeletal and endocrine system [39].

**FIGURE 5:**
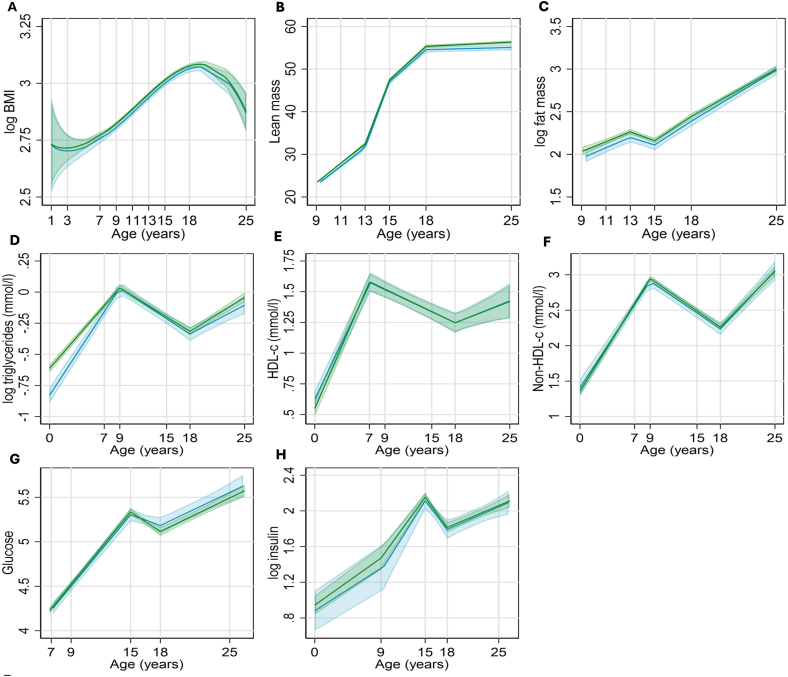
Predicted mean (95% CI) trajectories of cardiometabolic risk factors (DXA-measured lean mass and fat mass, BMI, and fasting blood samples) of preterm (blue) and full-term (green) births in the Avon Longitudinal Study of Parents and Children United Kingdom birth cohort (*n* = 311–676; range, 25–36 wk of gestation) and term (*n* = 4973–10,534). Reproduced with permission from reference [41]. CI, confidence interval; DXA, dual-energy X-ray absorptiometry.

**FIGURE 6:**
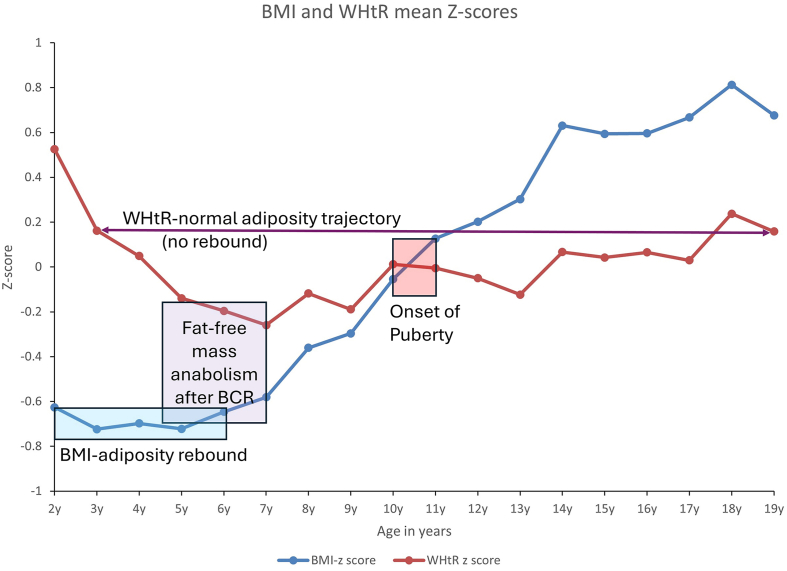
The NHANES 2021–2023 cycle, including 2–19 y olds (*n* = 2410). Supposed BMI-adiposity rebound in blue was observed at age 2 y when mean BMI Z-score was –0.63 and at age 6 y when the mean BMI Z-score was –0.64. WHtR mean Z-score at age 2 y was 0.53, and WHtR mean Z-score at age 18 y was 0.24. WHtR-adiposity rebound in purple does not exist until after ≥16-y period. The mean WHtR Z-score at age 3 y was 0.16 and at age 19 y was 0.16. The mismatch between BMI-adiposity rebound and WHtR-adiposity physiologic trajectory in purple suggests a fat-free mass (skeletal muscle mass) anabolism after a BCR. Age 10–11 is the mean age of puberty onset, which corresponds to the intersection of BMI and WHtR Z-score trajectories. BCR, body composition reset; WHtR, waist circumference-to-height ratio.

Another observation is that the linear increase in BMI from ages 6–18 y does not highlight the role of puberty-induced metabolic and hormonal changes during growth from childhood into adolescence, which significantly affect fat mass [40,41]. WHtR trajectories decreased between ages 11 and 13 y, which is the onset of puberty, and also around age 14–17 y, which is the end of puberty. These trajectories correspond to DXA-fat mass, lipids, glucose, and insulin concentration trajectories during and after puberty, already published in several prospective epidemiological studies and randomized clinical trials in the young population (FIGURE 5, FIGURE 6) [9,[41], [42], [43]]. Pubertal maturation alters the relative proportions of muscle, water, fat, and bone via the influence of the gonadal steroid hormones and growth hormones [44]. Due to testosterone, males increase muscle mass with simultaneous fat loss, reaching a zenith during the time of peak height velocity [44]. Decreasing height velocity postpuberty increases fat accumulation in both males and females, but twice as rapidly in the latter [44]. This change in DXA-measured body composition, especially regarding adiposity, seems consistent with another important observation that children ages 2 through age 5 y have a significantly high WHtR estimated fat mass similar to values in late adolescence and early adulthood. This might suggest a physiological adaptation to life, cellular and metabolic function, and survival [45].

It is known that children have 4 times more lean mass than fat mass, which decreases to 3 times more skeletal lean mass than fat mass by young adulthood (mid-twenties) [8,11,43]. At birth (36 wk of gestation), Ziegler et al. [46] reported in 1976 that babies had 0.24 kg of fat mass and 2.46 kg of fat-free mass on average. Demerath et al. [47] reported that 36-wk-old babies had an mean of 0.28 kg of fat mass and 2.41 kg of fat-free mass measured with air displacement plethysmography. This suggests that at birth, babies might have 10 times more fat-free mass than fat mass, with a significant percentage being water. This physiologic body composition profile invalidates BMI-adiposity rebound since BMI overwhelmingly measures fat-free mass rather than fat mass in early childhood [11]. BMI is a poor proxy for relative fatness in neonates, and De Cunto et al. [48] found that in term infants measured at birth, BMI explained only 43% of the variation in fat mass and had high estimation errors. The drop in BMI after age 1 y, as reported 42 y ago, could describe a reduction in fat-free mass, which was regained in a catch-up growth post 4 y of age (Figure 6) [1].

**FIGURE 7:**
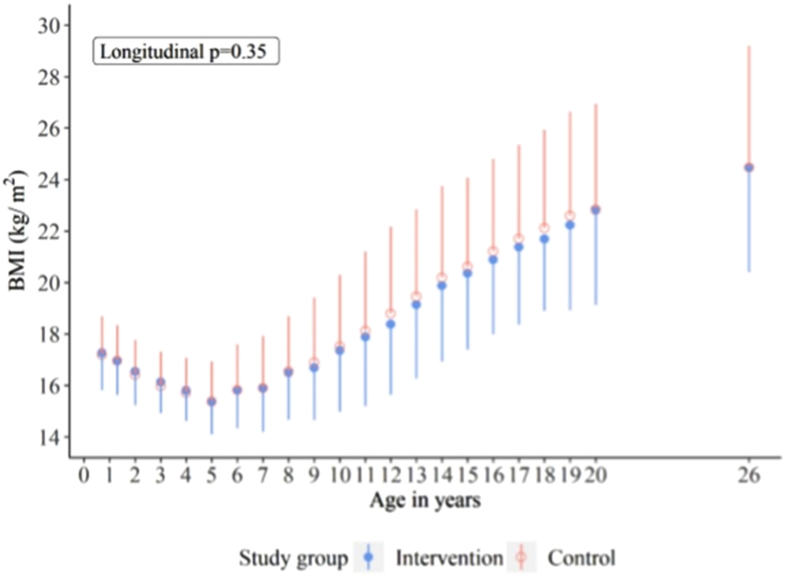
Mean (SD) BMI in the STRIP intervention and postintervention study conducted among 1116 infants in Finland from age 7 mo until age 26 y. Reproduced with permission from reference [42]. STRIP, Special Turku Coronary Risk Factor Intervention Project.

An unnecessary under-5 obesity prevention intervention facilitated by BMI-*adiposity rebound* theory may distort a normal physiologic process, i.e., the BCR effect, leading to poor lean mass accumulation with subsequent pubertal and hormonal alterations [44]. We and others have shown that optimal muscle mass in youth is cardioprotective and also has metabolic health benefits, such as decreasing the risk of insulin resistance, hyperglycemia, fat mass obesity, and type 2 diabetes [[7], [8], [9], [10]]. A 20-y-long repeated dietary counseling randomized clinical trial conducted among 1116 Finnish infants aged 7 mo, with an additional 6-y postintervention evaluation, revealed that BMI-adiposity rebound did not significantly differ between the intervention and control group (Figure 7) [42]. However, there was a significant reduction in poor cardiometabolic indices such as dyslipidemia, insulin resistance, and elevated blood pressure, in the intervention group compared with the control [42]. It is well established that poor cardiometabolic indices are risk factors for obesity and premature structural and functional heart damage in youth [9,[49], [50], [51]]. The failure of a 20-y-long randomized controlled trial to alter BMI trajectory, especially *adiposity rebound,* strongly suggests that BMI-*adiposity rebound* is not a deviation from normal childhood growth and development that should be corrected or reversed with early dietary interventions, but largely a statistically created anomaly [12,42].

A healthy diet introduced between the ages 4 and 7 y may significantly contribute to the body composition, neurologic, and cardiometabolic health of a growing child. Socioeconomic deprivations that significantly reduce the availability of healthy nutrition might be addressed if government agencies and policymakers provide well-nourished school meals for all children between the ages of 4 y and at least age 7 y, when the BCR effect and the fat-free mass anabolism mechanism kick start. Among 14,121 youths aged 5–18 y, the United States Healthy, Hunger-Free Kids Act of 2010, which mandated improvements in the nutritional quality of school meals and snacks, was associated with a reduced BMI in children and adolescents [52]. A systematic review of 6 studies comprising >11,000 schools in the United States concluded that universal free school meals were associated with increased meal participation and potentially increased attendance, and decreased obesity prevalence [53]. In a recent cohort study of 1052 schools in the United States, including 155,778 participants aged 4–18 y, school provision of universal free meals was associated with an 11% net reduction in the proportion of patients with high blood pressure measurements [54]. In a nutshell, evaluating the impact of school meals on BMI outcomes can be misleading, as a postintervention decrease in BMI could refer to both loss of fat mass and fat-free mass [11,43,54]. The latter is crucial for healthy growth and maturation; hence, WHtR might be a better adiposity surrogate outcome to detect the effectiveness of dietary intervention [10,[55], [56], [57], [58]].

### Limitations of the accuracy of new pediatric WHtR cutpoints in children <5

**FIGURE 8:**
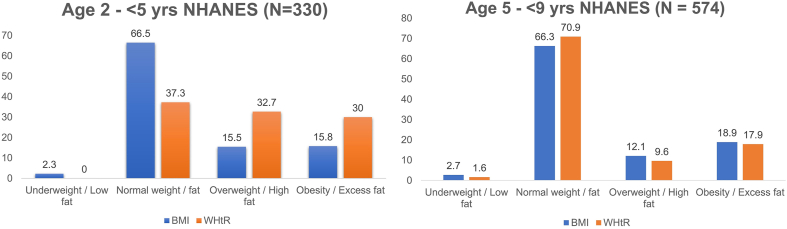
Under 5 y olds have higher fat mass than >5 y olds, suggestive of physiological adaptation for early childhood survival and metabolic cell function and regulation. WHtR, waist circumference-to-height ratio.

Although a new pediatric WHtR cutpoint has been established to accurately detect excess adiposity in the young population, significantly better than BMI, children aged <5 y appear to have a high WHtR-assessed fat mass similar to values in young adulthood [4,11,28,58]. Hence, the newly derived pediatric WHtR cutpoints cannot be used in children aged <5 y, as they will classify >60% of normal and healthy <5’s as having high and excess fat, twice as much as BMI estimates (Figure 8). The American Academy of Pediatrics has identified other surrogate and universally accessible adiposity markers apart from BMI as a gap in obesity clinical practice guidelines, but utilizing a single WHtR cutoff to accurately identify all pediatric participants will lead to misdiagnosis [3]. Therefore, age and sex-specific WHtR cutoffs are required for ages 2, 3, and 4 y before the future update of clinical guidelines on pediatric obesity.

In addition, future pediatric clinical guidelines may remove targeting *adiposity rebound* as a preventive strategy for obesity prevention because it is a statistical artifact. In a recent pediatric study, BMI overestimated the prevalence of overweight 3-fold and significantly underestimated underweight when compared with WHtR estimated fat mass [4]. Longitudinal studies and randomized clinical trials [4,11,23,24,28,30,31,59], among adults and the pediatric population, have established WHtR as a specific adiposity marker that overcomes BMI limitations in accurately diagnosing cardiometabolic diseases by distinguishing lean mass from fat mass and being relatively stable during growth from childhood, compared with the large BMI variability. Hence, WHtR should be universally adopted in the pediatric population as a specific metric for excess adiposity detection, screening, diagnosis, and management. Nonetheless, BMI at severe obesity range (>35 kg/m^2^) might have similar accuracy and sensitivity as the WHtR-excess fat mass cutpoint [3,4,11,28,59].

### Knowledge gaps and future direction

It is important to validate the new evidence on BCR effect and fat-free mass anabolism in a lean cohort, potentially from Europe or Asia. The mean WHtR during growth from ages 9 through 13 y in a British cohort [11] was consistently 0.45, whereas children of similar age in the United States NHANES 2021–2023 cycle cohorts had a mean WHtR value of 0.49. The mean lean mass and total fat mass in a 9-y-old British child are 24.5 and 8.6 kg, respectively [11,60]. However, in the United States cohort, the mean lean mass and total fat mass in a 9-y-old child are 23.6 and 11.9 kg, respectively. This suggests that a 9-y-old United States child has >3 kg more total fat mass than a British child, hence, the significant difference in WHtR, an excess of 0.04. Because the amount of lean mass in both United States and British children is similar, the fat-free mass anabolism physiological explanation could be generalized to other European cohorts. A major challenge to overcome is that several available datasets in the pediatric population across the globe lack WHtR measures from age 2 y through adolescence, since recommendations to measure WHtR as an adiposity marker are lacking in current clinical practice guidelines [3]. Future data collection during routine health surveys, cross-sectional studies, prospective studies, and clinical trials on body composition and anthropometry in children and adolescents must include WHtR assessments, even among infants. The lack of WHtR data will continue to reinforce the BMI-false narratives in the growing child, leading to misdiagnosis, mislabeling, and waste of scarce health resources [4] The WHO and the United States Center for Disease Control and Prevention are strongly urged to publish WHtR charts for children and adolescents globally, whereas the United Kingdom NICE obesity guidelines need an update to reflect pediatric WHtR cutpoints [61].

The stability of WHtR during growth from childhood through adolescence makes it a perfect surrogate measure of excess adiposity that must be universally adopted. WHtR does not significantly vary with age and sex, and distinguishes fat mass from lean mass, which is the major limitation of BMI [4,11,28]. Novel WHtR pediatric adiposity cutpoints have been validated as a predictor of type 2 diabetes, liver steatosis, and bone fracture risks in multiracial adult and young populations [4,11,28,29]. Future studies are warranted to longitudinally validate these WHtR pediatric cutpoints in predicting hypertension, inflammation, premature kidney damage, vascular dysfunction, premature cardiac damage, metabolic syndrome, neurodegenerative diseases, and mortality in both clinical and healthy population. This evidence will be sine qua non to updating future clinical practice guidelines on pediatric obesity screening, diagnosis, and management [3].

Three separate Cochrane systematic reviews and meta-analyses recently synthesized several hundred dietary and physical activity randomized controlled trials conducted among 2–4 y olds, 5–11 y olds, and 12–18 y olds globally, and concluded that the interventions had little to no effect in reducing childhood and adolescent obesity [[56], [57], [58]]. These unexpected and disappointing findings largely stem from a wrong choice of measurement outcome (BMI) in growing children, leaving childhood obesity prevention evidence in limbo [3]. We have shown that physical activity longitudinally increases lean mass, and because BMI reflects both lean mass and fat mass, it is difficult to observe a reduction in fat mass while employing BMI as an outcome, given that children may have 2–4 times more lean mass than fat mass, depending on their geographic locations [3,11,43]. Therefore, it is strongly recommended that all future clinical trials aimed at preventing and treating childhood obesity should utilize a better adiposity surrogate outcome, WHtR, from 2026 onward, rather than reinventing the wheel.

Another BMI-based controversy is “Metabolically Healthy Obesity (MHO)” first described by Jean Vague and published in the *American Journal of Clinical Nutrition* in 1956 (70 y ago) [62]. Incidentally, Vague proposed MHO in 1956, and Rolland-Cachera proposed *adiposity rebound* in 1984, and both are French scientists based in France [1,62]. Vague observed that a subgroup of people with obesity did not exhibit overt cardiometabolic abnormalities [62]. The diagnostic criterion for MHO is BMI ≥30 kg/m^2^ and absence of dyslipidemia, hypertension, and/or dysglycemia [63]. Furthermore, evidence suggests that MHO is characterized by reduced liver fat, visceral fat and inflammation, insulin sensitivity, increased subcutaneous leg fat content, normal adipose tissue function, and higher cardiorespiratory fitness and physical activity [[63], [64], [65]]. Applying different dyslipidemia or hypertension cutoffs doubled MHO prevalence in both adults and children [63,66]. Genetic data suggest that adipose tissue distribution and expansion, especially in the gluteofemoral adipose tissue compartment, is a determinant of MHO [66]. It is known that WHtR is 6 times better at detecting excess liver fat, a better predictor of type 2 diabetes and CVDs compared with BMI [4,24,25,28,30]. Moreover, the characteristics of MHO resonate with a healthy and active skeletal muscle, enhancing a metabolic homeostatic capacity and cardiorespiratory function [10,[63], [64], [65],^67^]. Because BMI cannot distinguish lean mass from fat mass, the term MHO might be an epiphenomenon secondary to the wrong adiposity assessment tool. Of note, neither WHtR nor DXA was available to Jean Vague 70 y ago [62]. It is likely that if WHtR is used in categorizing excess adiposity, MHO may not exist, given that WHtR is an accurate tool for detecting both total fat mass and trunk fat mass [11]. Moreover, hypertension is a disease diagnosed with an accurate measurement tool, and there is no such clinical diagnosis as “metabolically healthy hypertension.” Therefore, future research is needed to confirm or refute the hypothesis that MHO is a BMI-induced artifact, similar to *adiposity rebound* and the “obesity paradox.” [67].

Lastly, new obesity therapies such as glucagon-like peptide-1 receptor agonists (GLP-1 RAs) have been reported to have an unintended drawback, with patients reporting significant loss of muscle mass of ∼40%, potentially exacerbating or precipitating sarcopenic obesity [68,69]. These clinical trials have employed BMI as an adiposity primary outcome. For example, a systematic review and meta-analysis of 18 clinical trials (11 in obesity, 6 in type 2 diabetes, and 1 in prediabetes) with 1402 participants (838 GLP-1 RA users compared with 564 placebo) among 6–17 y olds concluded that GLP-1 RAs significantly improved glycemic, weight, and cardiometabolic outcomes [70]. A weight reduction of –3 kg and BMI reduction of –1.45 kg/m^2^ was reported, but there was no assessment of fat mass or WHtR [70]. The 3 kg weight reduction may significantly include lean mass, but the study duration of 0.5–1 y might not be sufficient to identify adverse consequences of lean mass loss in the young population. Considering that skeletal muscle mass anabolism is critical to metabolic function and pubertal development among children and adolescents, GLP-1 RAs may pose more unintended risk to the young population with obesity; therefore, long-term clinical trials and follow-up studies are urgently warranted to establish the lean mass-preserving ability of these drugs [[68], [69], [70]]. A recent study involving >400,000 middle-aged adults reported that patients who use GLP-1RAs have an increased risk of chronic cough [71]. Respiratory muscle atrophy, chest wall remodeling, and altered fiber type and metabolism are risk factors for chronic pulmonary diseases; however, whether lean mass loss explains persistent chronic cough remains unexamined [3,71,72]. Nonetheless, WHtR measured adiposity outcome could be a relevant primary outcome in GLP-1 RAs clinical trials in the young population [3,70].

In conclusion, data analysis of the United States NHANES 2021–2023 pediatric cohort highlights the potential erroneous interpretation of previously observed BMI-*adiposity rebound* in children. This study concludes that a better adiposity surrogate marker (WHtR) failed to confirm the existence of a similar *adiposity rebound.* Although mean values of BMI at age 2 y were attained by age 6 y after a significant decrease, WHtR values at age 2 y were never attained throughout childhood and adolescence beyond age 19 y. However, WHtR values at age 3 y seem to be regained at age 19 y, ∼16 y later. *Adiposity rebound*, which was coined 42 y ago, is not physiologic; rather, it is an epiphenomenon [12,13]. It is a BMI-induced false discovery similar to the “obesity paradox” in adults [25]. Fat-free mass anabolism is likely the accurate physiologic explanation that occurs beyond the BCR effect, starting after age 4 y. Science is self-correcting; it might be high time we updated pediatric obesity literature on the misleading BMI-*adiposity rebound* critical period and finally lay this statistical artifact to rest.

## Author contributions

The author's responsibilities were as follows– AOA: concept, design, and acquisition of data; AOA: statistical analysis; AOA: initial drafting of the manuscript; AOA: interpretation of data, critical revision, and finalization of the manuscript for important intellectual content; AOA: obtained funding. This publication is the work of the authors, and AOA will serve as guarantor for the contents of this paper. AOA had full access to all the data in the study and take responsibility for the integrity of the data and the accuracy of the data analysis; and read and approved the final manuscript.

## Funding

Agbaje’s research group (UndeRstanding FITness and Cardiometabolic Health In Little Darlings: urFIT-child) was funded by the Jenny and Antti Wihuri Foundation (grant no: 00180006), the North Savo regional and central Finnish Cultural Foundation (grants no: 65191835, 00200150, 00230190, and 00250189), Orion Research Foundation sr, Aarne Koskelo Foundation, Antti and Tyyne Soininen Foundation, Kuopio University Foundation, Paulo Foundation, Paavo Nurmi Foundation, Yrjö Jahnsson Foundation (grant no: 20217390), Ida Montin Foundation (grant no: 20230289), Fund of Eino Räsänen and Fund of Matti and Vappu Maukonen via the Faculty of Health Sciences University of Eastern Finland Pediatric Research Foundation, the Finnish Foundationfor Cardiovascular Research, (grant nos. 220021, 230012, and 240003), the Alfred Kordelin Foundation (230082), the 2024 European Association for the Study of Obesity-Novo Nordisk Foundation New Investigator Award for Childhood Obesity (NNF24SA0090437), and the American Society for Nutrition Foundation - Novo Nordisk Foundation Flemming Quaade Award for Innovative Approaches in Childhood Obesity (NNF25SA0104079). The funders had no role in the design and conduct of the study; collection, management, analysis, and interpretation of the data; preparation, review, or approval of the manuscript; and decision to submit the manuscript for publication.

## Conflict of interest

AOA reports financial support was provided by Novo Nordisk Foundation. If there are other authors, they declare that they have no known competing financial interests or personal relationships that could have appeared to influence the work reported in this paper.
